# Recent advances and challenges in antibacterial drug development: Editorial

**DOI:** 10.5599/admet.1315

**Published:** 2022-03-04

**Authors:** Lynn Silver

**Affiliations:** LL Silver Consulting, New Jersey, United States; silverly@comcast.net

There is a continuing need for new antibacterial drugs to deal with the burgeoning of bacterial pathogens resistant to the existing armamentarium of antibacterial drugs, compromising their effectiveness; furthermore, emerging pathogens are now considered to be a major microbiologic public health threat. Over the years, these issues have been addressed with the modification of existing antibiotic classes or the search for new structural classes. The latter approach has not been particularly successful, as evidenced by the fact that the last novel antibacterial class to be developed was discovered in 1984. Today the clinical pipeline is predominantly occupied by derivatives of established classes, although there are some novel entries in development [1].

Since the beginning of the century, Big Pharma began exiting antibacterial drug discovery despite a growing clinical need [2]. Apart from financial issues and regulatory hurdles, antibacterial discovery research, especially target-based approaches, has not been productive. The current *preclinical* pipeline has high diversity, with > 40 % of the projects representing new classes, new mechanisms of action or new targets [3]. However, this has generally been true of the preclinical pipeline all along (even when it was largely the domain of Big Pharma). Now, these projects are most often developed by SMEs residing in North America or Europe. The problem arises in the validation and successful development of these preclinical projects into actual clinical candidates – and that is where most projects fail. Clearly focused efforts toward overcoming the obstacles to antibacterial discovery are needed [4]. Research and resources are essential to progress novel approaches to clinical practices in order to sustainably fight against antibacterial resistance and emerging bacterial pathogens.

This special issue of ADMET and DMPK is guest-edited by Lynn Silver, LL Silver Consulting and Ana Budimir, University of Zagreb. Articles in this issue include a brief review of “Recent advances and challenges in antibacterial drug development” by Valeria Gigante et al. from the WHO [5] and a review of “Old and Modern Antibiotic Structures with Potential for Today’s Infections” by David Newman [6]. Guangshun Wang and A.F. Mechesso provide a “Realistic and critical review of the state of systemic antimicrobial peptides” [7]. In the area of new technologies, there is a review of “Recent advances in nanoparticles as antibacterial agents” by Murat Ozda and Sumeyra Gurkok [8]. Balbina Plotkin and Monika Konaklieva discuss the “Impact of host factors on susceptibility to antifungal agents” [9]. Matthias Fellner presents “Newly discovered Staphylococcus aureus serine hydrolase probe and drug targets” [10] and Andrei Bogdanov, et al. [11] describe a novel antibacterial scaffold in “Synthesis and diverse biological activity profile of triethylammonium isatin-3-hydrazones.”
